# Diagnostic pitfalls in systemic juvenile idiopathic arthritis: insights from 6 misdiagnosed cases

**DOI:** 10.3389/fped.2026.1885428

**Published:** 2026-08-17

**Authors:** Xiaona Zhu, Yinyin Guo, Jixin Guo, Jun Yang, Tingyan He

**Affiliations:** Department of Rheumatology and Immunology, Shenzhen Children’s Hospital Affiliated Shantou University Medical College, Shantou University, Shenzhen, China

**Keywords:** autoinflammation disease, diagnosis, inflammatory, misdiagnosis, systemic juvenile idiopathic arthritis

## Abstract

**Objective:**

Systemic juvenile idiopathic arthritis (sJIA) is a diagnosis of exclusion with nonspecific early manifestations. Misdiagnosis is common due to overlap with infections, autoinflammatory diseases, and malignancies. We aimed to characterize a series of patients initially diagnosed with sJIA who subsequently reclassified following further evaluation were.

**Methods:**

We retrospectively reviewed the clinical records, laboratory findings, genetic analyses, and imaging data of six pediatric patients initially diagnosed with sJIA but later revised to alternative diagnosis after further investigations. These patients were enrolled at Shenzhen Children's Hospital between January 2011 and December 2022.

**Results:**

All six patients initially fulfilled at least one set of sJIA classification criteria. All patients presented with recurrent fever (100%, 6/6), three had arthritis (50%, 3/6), and five exhibited rash (83.3%, 5/6). Elevated inflammatory markers were observed in all patients (100%, 6/6). The median age at initial sJIA diagnosis was 64 months (IQR, 24–145). The median delay from initial diagnosis to final diagnosis was 9.5 months (IQR, 5–12). Three patients (50%, 3/6) were considered to have refractory sJIA prior to diagnostic revision. Final diagnoses included autoinflammatory diseases (*n* = 3), Takayasu arteritis (*n* = 1), inflammatory myofibroblastic tumor (*n* = 1), and suspected congenital hemophagocytic lymphohistiocytosis (*n* = 1).

**Conclusions:**

sJIA is defined by classification criteria and represents a diagnosis of exclusion, requiring long-term dynamic reassessment to distinguish it from other diseases with overlapping clinical features. Early recognition of atypical manifestations and timely comprehensive evaluation are essential to avoid misdiagnosis, particularly in distinguishing monogenic autoinflammatory disorders and malignancies.

## Introduction

Juvenile idiopathic arthritis (JIA) is a heterogeneous group of chronic inflammatory diseases of unknown etiology with onset before the age of 16 years (1). Systemic juvenile idiopathic arthritis (sJIA) is a distinct subtype of JIA, characterized by markedly elevated inflammatory markers, recurrent fever, and evanescent rash. It accounts for approximately 10% of JIA cases in the Western world and appears to be more common in Asia populations. The reported incidence and prevalence of sJIA were 0.6 (95% CI, 0.5–0.7) and 3.1 (95% CI, 2.7–3.6) per 100,000 children, respectively, with a median age at disease onset of approximately 6.4 years (IQR, 3.4–10 years) (2). Arthritis may be absent at disease onset and develop later during the disease course. Additional features, including lymphadenopathy, hepatosplenomegaly, and serositis may be present, and some patients may develop life-threatening macrophage activation syndrome (MAS) (3).

The pathogenesis of sJIA is primarily driven by dysregulation of the innate immune system, with excessive production of proinflammatory cytokines such as interleukin (IL) -1, IL-6, and IL-18 (4). However, there are no disease-specific laboratory biomarkers, and the diagnosis largely relies on classification criteria, including those proposed by the International League of Associations for Rheumatology (ILAR) (5), the Pediatric Rheumatology International Trials Organization (PRINTO) (6) and the European Alliance of Associations for Rheumatology (EULAR) in collaboration with the Pediatric Rheumatology European Society (PReS) (7). Importantly, sJIA is fundamentally a diagnosis of exclusion, requiring careful evaluation to rule out alternative conditions.

A wide range of diseases, including malignancies, infections, and other autoimmune or autoinflammatory diseases (8–12), may present with overlapping features such as fever, rash, elevated inflammatory markers, and organomegaly. This clinical overlap poses a significant diagnostic challenge, particularly in the early stages of disease. Although several case reports have described individual diseases that mimic sJIA, a practical summary of common diagnostic pitfalls and clinical warning signs remains lacking. To address this gap, we present a case series of six pediatric patients who initially fulfilled sJIA classification criteria but were ultimately diagnosed with other conditions following comprehensive re-evaluation. Beyond describing these cases, our study summarizes practical clinical red flags and proposes a diagnostic algorithm to facilitate timely diagnostic reassessment and improve recognition of sJIA mimickers in routine clinical practice.

## Methods

### Patient cohort and study approval

This retrospective observational descriptive case series included six pediatric patients who were initially diagnosed with sJIA at the Department of Rheumatology and Immunology, Shenzhen Children's Hospital, between January 2011 and December 2022. All patients were subsequently reclassified with alternative diagnoses following comprehensive diagnostic evaluation. The inclusion criteria were as follows: (1) newly diagnosed patients fulfilling the clinical classification criteria for sJIA according to the ILAR 2001 (5) or the PRINTO 2019 (6) or the EULAR/PReS 2024 (7); (2) availability of further diagnostic investigations, including genetic testing, pathological examination, vascular imaging and etiological assessments, allowing confirmation of the final diagnosis; and (3) complete clinical data, including medical history, physical examination findings, laboratory and imaging results, treatment course, and follow-up outcomes. The study involving human participants were reviewed and approved by the Ethics Committee of Shenzhen Children's Hospital (201903807). Because this retrospective study involved anonymized clinical data with minimal risk, the requirement for written informed consent was waived by the Ethics Committee.

### Data collection

Clinical data were collected from inpatient medical records, including demographic characteristics, clinical manifestations, and laboratory findings. Laboratory parameters included leukocyte count, neutrophil count, platelet count, hemoglobin level, erythrocyte sedimentation rate (ESR), C-reactive protein (CRP), ferritin, and fibrinogen levels. Serum cytokines profiles included interferon (IFN)-*γ*, IL-10, IL-6, tumor necrosis factor (TNF)-*α*, IL-4, and IL-2. Results from genetic testing, pathological biopsy and imaging examinations were collected and analyzed. Clinical data were independently extracted and reviewed by two investigators using a standardized data collection form. Discrepancies between reviewers were resolved by discussion and, when necessary, consultation with the senior corresponding author until consensus was achieved.

### Statistical analysis

Descriptive statistical analysis was performed. Continuous variables were expressed as median with interquartile ranges (IQR), and categorical variables were presented as counts and percentages. Statistical analysis was conducted using GraphPad Prism 8.0 statistical software (GraphPad Software Inc., La Jolla, CA, United States).

## Results

### Baseline clinical characteristics

A total of six patients were included, comprising five males and one female. The median age at disease onset was 63 months (IQR, 14–135), and the median age at initial sJIA diagnosis was 64 months (IQR, 24–145). All patients fulfilled the ILAR or PRINTO or EULAR/PReS classification criteria for sJIA. The median delay from initial diagnosis to final diagnosis was 9.5 months (IQR, 5–12) (Table 1).

**Table 1:** Baseline clinical characteristics of the six patients initially diagnosed with or suspected of sJIA.

Patient	Gender	Age at disease onset (months)	Age at initial diagnosis (months)	Age at final diagnosis (months)	Diagnostic delay (months)	Initial sJIA-like manifestations	Fever	Rash	Musculoskeletal involvement	Hepatomegaly	Splenomegaly	Lymphadenopathy	Serositis	Final diagnosis
1	Male	14	15	22	7	Fever, rash, hepatosplenomegaly,bilateral multiple parenchymal and interstitial lung changes	1	1	0	1	1	0	0	Suspected familial HLH
2	Male	35	36	48	12	Fever, rash, lymphadenopathy, recurrent oral ulcer	1	1	0	1	0	1	0	PFAPA
3	Male	91	92	94	2	Fever, panniculitis-like rash and mild coronary artery dilatation	1	1	0	0	0	0	0	Takayasu arteritis
4	Female	135	147	159	12	Fever, arthritis, anemia, abdominal symptoms	1	0	1	1	1	0	0	Inflammatory myofibroblastic tumor
5	Male	0.3	24	40	16	Fever, rash, anemia, arthritis, abdominal symptoms	1	1	1	1	1	1	0	Mevalonate kinase deficiency
6	Male	145	145	150	5	Fever, rash, arthritis, lymphadenopathy	1	1	1	1	1	1	0	NLRP12-associated autoinflammatory disease

sJIA, systemic juvenile idiopathic arthritis; HLH, hemophagocytic lymphohistiocytosis; PFAPA, periodic fever, aphthous stomatitis, pharyngitis, adenitis syndrome; NLRP12, pyrin domain containing 12; 1 = Present; 0 = Absent.

**Figure 1:**
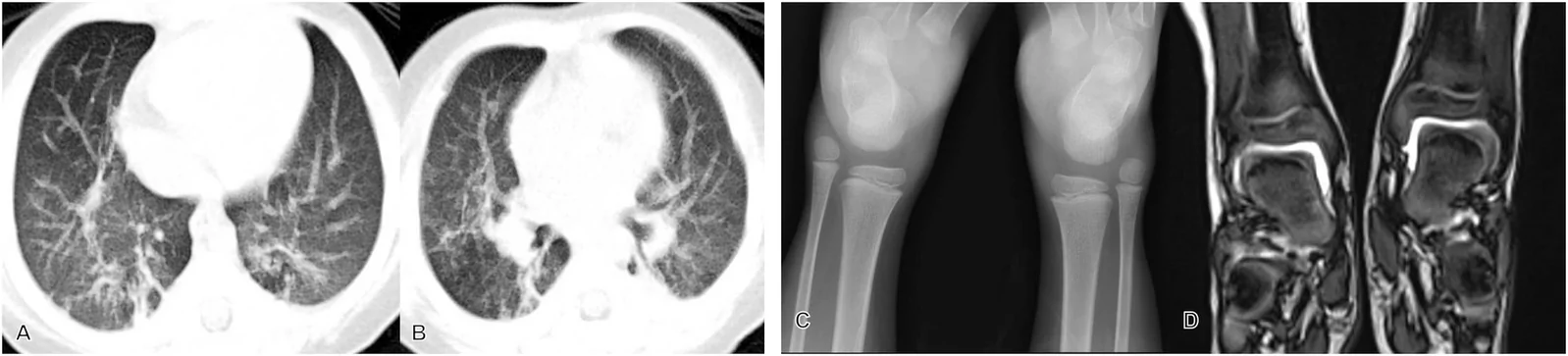
Imaging findings in patients 1 and 5. **(A,B)** Chest computed tomography (CT) images of patient 1 demonstrating bilateral pulmonary involvement with multiple parenchymal and interstitial lesions. **(C)** Plain radiographs of the ankles in patient 5 showing bilateral soft tissue swelling, with preserved joint surfaces and normal joint alignment. **(D)** Magnetic resonance imaging (MRI) of the ankles in Patient 5 revealing bilateral joint effusion and marked synovial thickening with contrast enhancement.

Fever was the initial manifestation in all patients (100%, 6/6). Five patients presented with skin rash (83.3%, 5/6). Except for panniculitis-like skin lesions observed in patient 3 (P3), all other patients exhibited evanescent erythematous rashes associated with fever, which resolved with defervescence. Three patients had musculoskeletal involvement at disease onset (50%, 3/6). Patient 4 (P4) presented with knee pain, while Patients 5 and 6 (P5 and P6) manifested with arthritis. P5 had migratory joint swelling, pain, and functional limitation, predominantly affecting the bilateral knees, ankles, elbows, and wrists. Magnetic resonance imaging (MRI) of the ankle joints demonstrated bilateral joint effusion with marked synovial thickening and enhancement (Figure 1D). P6 presented with pain and restricted movement of both knees and the interphalangeal joints of the hands. Hepatomegaly was observed in five patients (83.3%, 5/6), splenomegaly in four (66.7%, 4/6), and lymphadenopathy in three (50%, 3/6). Macrophage activation syndrome (MAS) was observed in three patients (P1, P2, and P6) (50%, 3/6).

Additional atypical features were noted in several patients. P1 had abnormal craniofacial features since birth and a history of cleft palate repair, consistent with Pierre Robin sequence. P2 had recurrent oral ulcers since the age of 6 months, with a positive maternal family history. P4 experienced intermittent abdominal pain accompanied by pallor. P5 had recurrent abdominal pain, a prior history of surgery for suppurative appendicitis, and episodes of intestinal obstruction.

### Laboratory and imaging findings

Inflammatory markers were markedly elevated in all patients (100%, 6/6), with median CRP, ESR, and ferritin levels of 88.2 mg/L (IQR, 57.7–120), 55 mm/h (IQR, 33–88), and 843.25 ng/mL (IQR, 311.93–1,248.7), respectively (Table 2). Anemia of varying severity was identified in three patients (P4, P5 and P6) (50%, 3/6), with patient 4 presenting with severe anemia. Serum cytokine analysis revealed that only P4 exhibited mildly elevated IL-6 levels, whereas no significant cytokine elevation was observed in the remaining patients.

**Table 2:** Laboratory and inflammatory features at initial sJIA-like presentation.

Patient	WBC (10^9^/L)	Neutrophils (10^9^/L)	Hemoglobin (g/L)	Platelets (10^9^/L)	CRP (mg/L)	ESR (mm/h)	Ferritin (ng/mL)	ALT (IU/L)	AST (IU/L)	Fibrinogen (g/L)	Triglycerides (mmol/L)	Lactate dehydrogenase (IU/L)	IL-6 (pg/mL)	IL-10 (pg/mL)	IFN-*γ* (pg/mL)
1	18.95	12.7	111	600	120	59	8,632	50	44	1.99	1.04	800	13.3	8.5	0.4
2	11.06	8.54	117	194	57.7	23	1,248.7	96	182	1.82	5.48	571	3.89	55.26	13.38
3	29.1	23.52	110	534	47	88	311.93	8	17	7.34	0.77	306	13.5	1.64	2.51
4	11.1	8.93	59	243	70.5	33	542	11	14	5.68	0.75	347	24.26	1.27	6.73
5	9.06	5.13	77	357	105.9	122	117.7	12	26	6.22	1.04	321	NA	NA	NA
6	30.64	28.5	102	366	236.6	51	1,144.5	26	42	4.5	1.17	555	NA	NA	NA

sJIA, systemic juvenile idiopathic arthritis; WBC, white blood cell; CRP, c-reactive protein; ESR, erythrocyte sedimentation rate; ALT, alanine aminotransferase; AST, aspartate aminotransferase; IL, interleukin; IFN, interferon; NA, not available.

**Figure 2:**
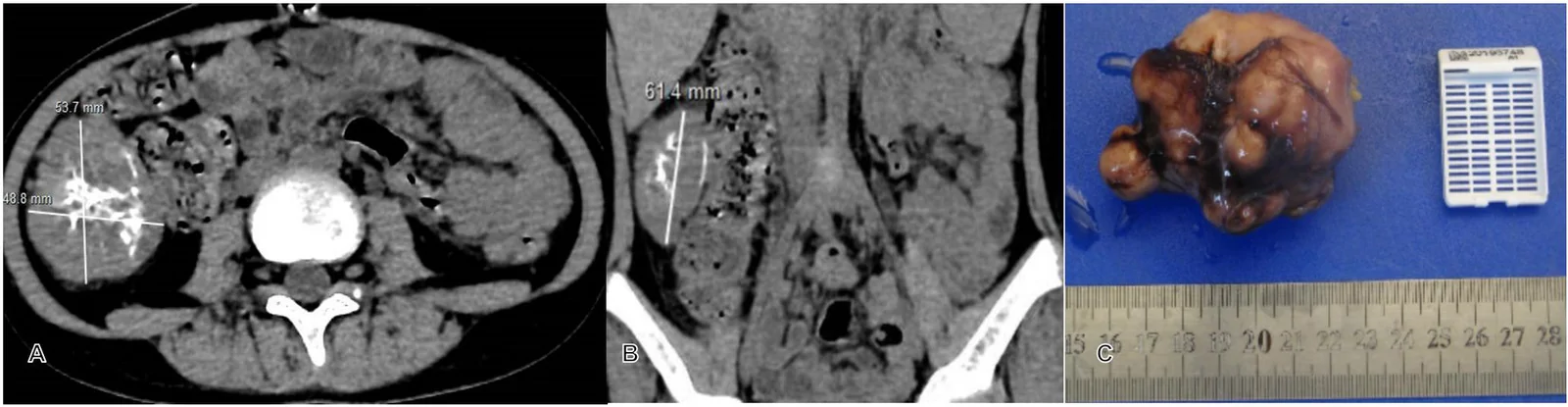
Abdominal imaging and surgical findings in patient 4. **(A,B)** Abdominal computed tomography (CT) images demonstrating a hypervascular mass located in the right upper abdomen, measuring approximately 49 × 54 × 61 mm, with multiple punctate and linear calcifications within the lesion. **(C)** Gross appearance of the surgically resected tumor specimen, showing a solid mass.

Imaging studies also revealed atypical features. Patient 3 demonstrated mild coronary artery dilatation, with a right coronary artery diameter of 3.1 mm (Z-score: 2.77) on echocardiography. P1 demonstrated extensive bilateral pulmonary involvement with multiple parenchymal and interstitial lesions (Figures 1A,B). Abdominal computed tomography (CT) in patient 4 identified a hypervascular mass in the right upper quadrant, with multiple punctate and strip-like calcifications, measuring approximately 49 × 54 × 61 mm (Figures 2A,B). Notably, the presence of coronary artery dilatation and an abdominal mass represented atypical findings for sJIA, raising suspicion for alternative diagnoses and prompting further diagnostic investigation.

### Treatment response and diagnostic reclassification

P1 presented with persistent hyperferritinemia. Despite treatment with the HLH-2014 protocol (dexamethasone combined with etoposide), the patient continued to experience recurrent thrombocytopenia and elevated ferritin levels. Although no pathogenic variants associated with primary hemophagocytic lymphohistiocytosis (pHLH) were identified, the early disease onset and poor treatment response supported a final diagnosis of suspected familial HLH.

P2 achieved disease remission following treatment with glucocorticoids (total duration: 3 months) combined with tocilizumab (total duration: 6 months). However, recurrent episodes of fever and tonsillitis occurred after treatment discontinuation. Given a history of recurrent oral ulcers during the early disease course and positive family history, the final diagnosis was revised to periodic fever, aphthous stomatitis, pharyngitis, and adenitis (PFAPA) syndrome. The patient is currently managed with vitamin D and cimetidine.

P3 developed progression of coronary artery dilation during glucocorticoid tapering. Follow-up vascular ultrasound revealed multi-vessel involvement consistent with vasculitis. Accordingly, the diagnosis was therefore revised to Takayasu arteritis, and treatment regimen was adjusted to cyclophosphamide therapy.

P4 experienced recurrent fever and persistent anemia despite multiple courses of glucocorticoids and tocilizumab treatment. Abdominal computed tomography (CT) revealed a mass lesion, and the patient subsequently underwent surgical resection of a greater omental tumor (Figure 2C). Histopathology examination confirmed an inflammatory myofibroblastic tumor.

P5 showed difficulty in glucocorticoids tapering, with recurrent hyperferritinemia and intermittent fever during dose reduction. Genetic testing identified a heterozygous NLRP12 variant (c.910C >T), which is extremely rare in the general population and has been previously reported in association with NLRP12-associated autoinflammatory disease (NLRP12-AID).

P6 presented with early-onset disease characterized by prominent abdominal pain, recurrent fever, rash, and arthritis. Genetic analysis revealed compound heterozygous variants in the mevalonate kinase (MVK) gene (c.827A >G and c.1127G >A), confirming a diagnosis of mevalonate kinase deficiency (MKD). Functional validation further demonstrated significantly reduced MVK enzyme activity, confirming the pathogenicity of these variants. Following discharge, the patient remained on low-dose oral prednisone (5–10 mg/day). During follow-up, fever, abdominal pain, and arthritis remained well controlled, anemia improved markedly, and the patient resumed normal school attendance.

## Discussion

Systemic juvenile idiopathic arthritis is a rare, heterogeneous autoinflammatory disorder characterized by prominent systemic inflammation. Despite the development of multiple classification criteria to standardize diagnostic assessment, sJIA remains fundamentally a diagnosis of exclusion, as no single pathognomonic biomarker has yet been identified (13). The ILAR criteria mandate persistent arthritis for at least 6 weeks, which may delay diagnosis in patients presenting predominantly with systemic manifestations. In contrast, the PRINTO criteria remove the requirement for arthritis and have been shown to improve early diagnostic sensitivity (80.5% vs. 62.2% for ILAR) while maintaining comparable specificity (90.5% vs. 91.0%, respectively) (14). The 2024 EULAR/PReS recommendations further unify the diagnostic framework for sJIA and adult-onset Still's disease (AOSD), recognizing them as a single disease spectrum with shared pathophysiological mechanisms.

Nevertheless, all current classification systems carry an inherent risk of misdiagnosis, particularly during the early disease course when clinical and laboratory manifestations are often non-specific. The high misdiagnosis rate of sJIA can be attributed to several factors. First, its core clinical features (fever, rash, and elevated inflammatory markers) are nonspecific and widely overlap with those of multiple mimicking conditions (15, 16). For example, both sJIA and familial HLH may present with persistent fever, hepatosplenomegaly, and markedly elevated ferritin levels. Similarly, monogenic autoinflammatory diseases (17) often manifest with recurrent fever and systemic inflammation, closely resembling early-stage sJIA. Second, limited awareness of rare differential diagnoses, such as inflammatory myofibroblastic tumor and monogenic autoinflammatory diseases, may further contribute to misdiagnosis, particularly in primary care settings where access to advanced investigations (including genetic testing or histopathology) is restricted.

In this study, all patients initially fulfilled established classification criteria for sJIA but were ultimately diagnosed with a spectrum of alternative conditions, including familial HLH, PFAPA syndrome, Takayasu arteritis, inflammatory myofibroblastic tumor, and monogenic autoinflammatory diseases such as NLRP12-AID and MKD. These findings highlight the substantial phenotypic overlap between sJIA and a heterogeneous group of inflammatory, genetic, and neoplastic disorders, underscoring the limitations of relying solely on classification criteria.

Based on our observations, clinicians should maintain a high index of suspicion for alternative diagnoses in patients presenting with sJIA-like features, particularly those with very early disease onset, atypical systemic manifestations, persistent or progressive laboratory or imaging findings, or an inadequate response to standard therapy. Notably, two patients in our cohort developed disease before 2 years of age, younger than the typical onset onset of sJIA (3–5 years) (1). Therefore, early genetic testing should be considered in infants and young children with an sJIA-like phenotype to evaluate for monogenic autoinflammatory diseases. In addition, most patients in our series experienced glucocorticoid dependence or an unsatisfactory response to tocilizumab. Given that approximately 60%–70% of patients with true sJIA achieve inactive disease status or low disease activity following IL-6 inhibitor therapy (18), failure to achieve meaningful clinical improvement should raise suspicion for an alternative diagnosis. Although no guideline defines an exact time point for diagnostic revision, current treat-to-target recommendations provide a practical framework for reassessment. The German PRO-Kind initiative recommends achieving at least 50% improvement by approximately 4 weeks and clinically inactive disease without glucocorticoids within 6 months (19), while the 2024 EULAR/PReS recommendations emphasize maintaining glucocorticoid-free clinically inactive disease for 3–6 months before tapering biologic therapy (7). We therefore suggest careful diagnostic reassessment when treatment targets are not achieved by approximately 4 weeks, or when patients remain glucocorticoid-dependent or fail to achieve clinically inactive disease within 3–6 months.

**Figure 3:**
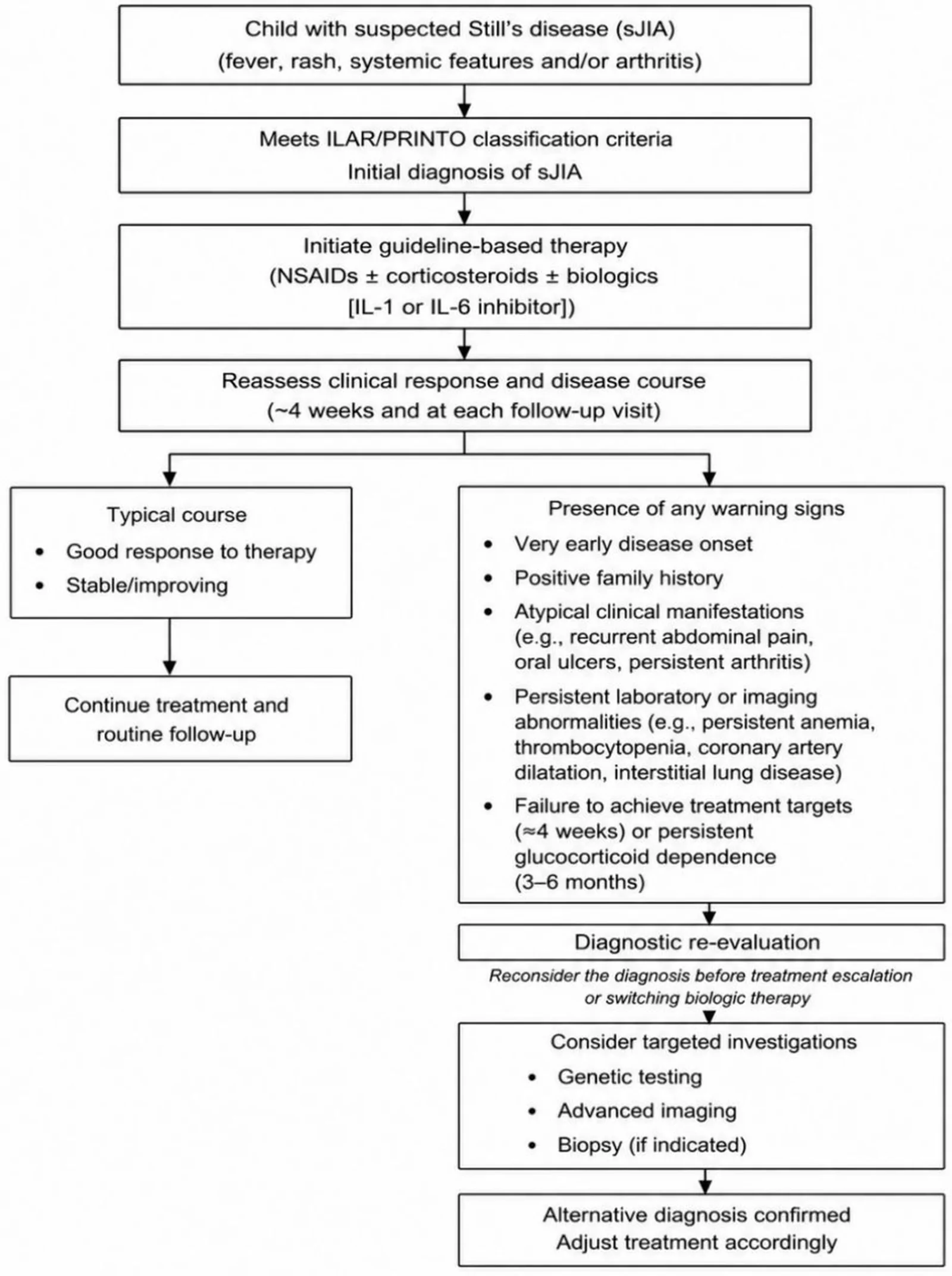
Proposed diagnostic algorithm highlighting clinical “red flags” that should prompt diagnostic reassessment in children initially diagnosed with systemic juvenile idiopathic arthritis (sJIA).

Furthermore, although abnormalities such as anemia and coronary artery dilatation have occasionally been reported in sJIA (20), they are usually transient. Persistent or progressive atypical clinical, laboratory, or imaging findings should therefore prompt further investigations for alternative diagnoses. Nevertheless, atypical manifestations alone do not exclude sJIA, as concurrent sJIA/AOSD and other pathological conditions have been reported (10). Diagnostic reassessment should therefore be comprehensive rather than leading to immediate exclusion of sJIA. Based on these observations, we propose a practical diagnostic algorithm (Figure 3) highlighting the major clinical “red flags” that warrant diagnostic re-evaluation.

This study has several limitations. First, this was a retrospective descriptive case series conducted at a single tertiary pediatric center with a small sample size, which may limit the generalizability of our findings to other institutions and patient populations. Second, the final diagnoses in some cases required specialized investigations, including genetic testing and pathological biopsy, and variability in diagnostic resources across institutions may influence the broader applicability of the results. In addition, we did not perform a quantitative comparative analysis of laboratory parameters between sJIA and its differential diagnoses.

Despite these limitations, this case series provides real-world evidence highlighting common diagnostic pitfalls in sJIA and offers practical clinical clues to assist clinicians in distinguishing sJIA from its mimickers in routine practice. These findings may ultimately contribute to improved diagnostic accuracy and more appropriate clinical decision-making in children presenting with systemic inflammatory syndromes.

## Conclusions

In conclusion, although current classification criteria provide a valuable framework for the classification of sJIA, they should not be regarded as diagnostic criteria or used as a substitute for clinical judgment. As a diagnosis of exclusion, sJIA requires continuous and dynamic reassessment to distinguish it from other diseases with overlapping clinical features, including inflammatory, genetic, and neoplastic diseases. Early recognition of atypical features, together with timely implementation of targeted investigations, including imaging and genetic testing, is crucial for improving diagnostic accuracy and optimizing patient outcomes.

## Data Availability

The original contributions presented in the study are included in the article/Supplementary Material, further inquiries can be directed to the corresponding author/s.
